# Electronic cigarette use and smoking cessation behavior among adolescents in China

**DOI:** 10.1016/j.addbeh.2018.02.029

**Published:** 2018-07

**Authors:** Xinsong Wang, Xiulan Zhang, Xiaoxin Xu, Ying Gao

**Affiliations:** School of Social Development and Public Policy, Beijing Normal University, Beijing, China

**Keywords:** Electronic cigarette, Cessation, Adolescents, Public policy, China

## Abstract

**Objective:**

China produces the majority of the world's electronic cigarettes (e-cigarettes) and e-cigarettes have become popular in the country, especially among young people. However, little is known about the characteristics of e-cigarette use in China and how it is associated with smoking cessation behavior. This study focuses on the adolescent group in China and examines their perception and use of e-cigarettes and the association with smoking abstinence.

**Methods:**

We use a mobile app-based survey on smoking behavior conducted in November 2015 in China, and focus on a sample of 2042 adolescents aged between 12 and 18. Awareness, perception, use of e-cigarettes are examined as well as the behaviors of promoting e-cigarettes and of smoking cessation. A logistic regression is performed to test the association between e-cigarette use and smoking abstinence behavior.

**Results:**

In 2015, nearly 90% of the surveyed adolescents in China were aware of e-cigarettes, while over a quarter of the respondents were ever users. The odds ratio for ever users of e-cigarettes to have tried to quit smoking conventional cigarettes was 1.60 that of never users. For those who tried to quit smoking, 36.02% indicated that they used e-cigarettes to help quit. However, only 13.52% of those who had used e-cigarettes to help quit smoking were successful in quitting.

**Conclusions:**

This study is one of the first empirical research on e-cigarette use among Chinese adolescents. E-cigarettes are widely known and quite popular among Chinese adolescents. As the association between e-cigarette use and smoking cessation behavior is less than clear, more empirical research is called for to help form evidence-based regulatory policy on e-cigarettes in China.

## Highlights

• Little is known about e-cigarette use among adolescents in China and how it is associated with smoking cessation behavior.
• Our 2015 survey on e-cigarette use and smoking cessation behavior covered 2042 Chinese adolescents aged between 12 and 18.
• E-cigarettes were widely known and popular among Chinese adolescents.
• There was a significant association between e-cigarette use and smoking cessation behavior.

## 1 Introduction

Electronic cigarette (hereafter abbreviated to e-cigarette), also known as the Electronic Nicotine Delivery System (ENDS), is a battery-powered smoking device that releases vaporized nicotine solution inhalable by users (Trtchounian & Talbot, 2011). Designed to mimic the appearance and feel of conventional combustible tobacco products, e-cigarette has been advertised and advocated as a healthier replacement for tobacco smoking due to its more flexible control of nicotine ingredients and releasing less toxicants than conventional cigarettes (Goniewicz et al., 2014). Adolescents are particularly susceptible to e-cigarette use as, among other reasons, it is fashionable and features various flavors (Durmowicz, 2014). Although research has demonstrated that e-cigarette may generate a number of toxicants and the impact of long-term use of e-cigarette use on health is uncertain (Vardavas et al., 2012), many smokers tend to use e-cigarettes as smoking cessation aids (Bullen et al., 2010; Eissenberg, 2010; Wang & Etter, 2004).

Is e-cigarette use helpful to smoking cessation? Current research findings are mixed. First of all, some research show that a significant portion of e-cigarette users did use it to quit smoking and regard e-cigarette as having the effect of aiding smoking abstinence (Etter, 2010). Second, some have demonstrated a positive effect of e-cigarette use on smoking cessation. A 6-month pilot study reveals that 55% of the smoking participants reduced the amount of smoking by 50% or abstained from smoking (Polosa et al., 2011). A more recent randomised-controlled trial finds that e-cigarette use modestly helped quitting although its impact may not be superior to that of nicotine patch (Bullen et al., 2013). Meanwhile, another study shows that smokers who used e-cigarettes were more likely to report continued abstinence than those who used nicotine replacement therapy (NRT) or those not using any cessation aid (Brown, Beard, Kotz, Michie, & West, 2014). Still another study of the short-term impact of e-cigarette use on smoking abstinence finds that it helped withhold the desire to smoke after 20 min of e-cigarette use (Dawkins, Turner, Hasna, & Soar, 2012). However, others have demonstrated that e-cigarette users were just as likely to be smoking as nonusers, raising doubts whether e-cigarette use has solid impact on smoking cessation (Borderud, Li, Burkhalter, Sheffer, & Ostroff, 2014).

Not only have the existing studies generated mixed results regarding the impacts of e-cigarette use on smoking cessation, very little research has studied the group of adolescents, whose e-cigarette use has intensified. Among the middle school and high school students in the U.S., the ever users of e-cigarettes more than doubled from 2011 to 2012 (from 3% to 7%) (Corey et al., 2013). In South Korea, 9.4% surveyed adolescents were ever users of e-cigarettes and 4.7% were current users, and the authors conclude that Korean adolescents were responding to e-cigarette advertisements that portrayed the products as smoking cessation aids (Lee, Grana, & Glantz, 2014).

While e-cigarette was invented and largely manufactured in China, research on the use of e-cigarette in the country has been extremely rare. A recent study of Hong Kong students shows that only 1.1% of the surveyed population reported having used e-cigarettes in the past 30 days (Wang, Ho, Leung, & Lam, 2015). Such statistics have not been available in mainland China, although e-cigarettes have become popular among the young population (Barboza, 2014). The China Global Youth Tobacco Survey (CGYTS) in 2014 provided some brief information on e-cigarette use but it covered those aged between 13 and 15 only (WHO, 2014).

Moreover, little is known whether e-cigarette use has any impact on smoking cessation among Chinese smokers. But the fact that such evidence is lacking seems to have convinced the State Tobacco Monopoly Administration (STMA) to promote e-cigarette in China as it may not necessarily affect the sales of conventional cigarettes, which is monopolized by the China National Tobacco Corporation, the corporate body of STMA (Zhang, 2015). If e-cigarette use does not help with smoking cessation, promoting it may risk renormalizing smoking in China, as ever users of e-cigarettes are more likely to become conventional cigarette users (Grana & Ling, 2014). If e-cigarette use does help with smoking cessation, however, the STMA may not have the motivation to promote it anymore. Examining smokers' attitude and behavior of using e-cigarettes as a cessation aid is very necessary in the world's largest cigarette consumer country. This research will focus on the adolescents' e-cigarette use and its association with their attempts of smoking abstinence in China.

## 2 Methods

We conducted a mobile internet survey to collect data on the attitude and behavior of cigarette and e-cigarette use. Previous tobacco research focusing on adolescents in China has relied on school-based surveys, which often are unable to reach the high schoolers in rural regions because many of them would enter the workforce after middle school. An internet survey should help us overcome the problem since adolescents of both urban and rural origins have access to the Internet. By June 2015, Internet users in China had reached 668 million or 48.8% of the total population, with 88.9% accessing the Internet through mobile devices. In both urban and rural regions, the majority of young population had access to the Internet. For example, among those aged between 10 and 19, 85.1% of urban and 65.6% of rural individuals had access to the Internet (CNNIC, 2015). Our questionnaires were distributed through the Kai-Di Community, a mobile-app based survey service with 152,622 registered app users, 91.75% of whom were under the age of 35. Among the app users, 53.1% were males and 46.9% were females. 46.1% of the app users had an education level of college and above, 33.5% had associate degrees, while 20.4% had a level of high school and below. In terms of occupation, the app users covered major professions including businesspersons (27.4%), service labor (20.2%), professionals (20.1%), and students (25.3%), although only a small percentage (6.9%) were government staff. The user locations approximately matched the population distribution across provinces in China, with the top five provinces where the app users were located being Guangdong, Sichuan, Shandong, Jiangsu, and Henan.

### 2.1 Study procedures and sample

This mobile app-based survey was conducted between November 16 and 18, 2015. Questionnaires were pushed to all registered mobile users who, if intending to respond, would have to fill out the questionnaires within 48 h. The app system recorded the time a respondent accessed the questionnaire, the exact time for each question to be answered, and the values filled in. All submitted questionnaires went through a logic check procedure performed by the system to check the completeness, social-demographic consistency with the registered information, and whether time spent on each question was reasonable (for example, the minimum time for each question was supposed to be 3 s). Once a questionnaire passed the system check, a second round of logic check was applied to the data with a focus on smoking history and perception and use of e-cigarettes. Finally, the data were derived from the system in Excel format. Each completed questionnaire was compensated for $0.80 to the respondent's account.

In total, 22,081 people under the age of 40 answered the questionnaires through the mobile app, and 17,663 or 80% of the respondents had complete and valid answers. Among them, 2042, or 11.56% were between the age of 12 and 18; 10,477, or 59.32% were aged between 19 and 29, and 5144, or 29.12% were aged between 30 and 39. This study analyzes the e-cigarette use and smoking abstinence among the 2042 adolescents.

### 2.2 Measures and statistical analysis

In general, we adopted the conventional measurements seen in existing studies about e-cigarettes and tobacco use. We divided the respondents into current smokers, former smokers and never smokers based on their answers to the question of smoking status (Reid, Rynard, Czoli, & Hammond, 2015). Current smokers were those who had smoked more than 100 cigarettes in total and were still smoking by the time of our survey. Former smokers were those who used to smoke but had quit by the time of our survey. Never smokers had smoked <100 cigarettes in their lifetime (Coleman et al., 2015). For current smokers, we asked their smoking frequency (daily or occasional) and how many cigarettes they smoked/day. Then we asked the current smokers whether they ever tried to quit smoking (yes vs no), and if “yes”, what methods they used to help with quitting. The options included “E-cigarettes”, “Patch or other products”, “Other methods” (Ramo, Young-Wolff, & Prochaska, 2015).

We measured the awareness of e-cigarettes with the question, “Have you heard of e-cigarettes (yes vs no) (Jiang et al., 2016)?” Those who answered “yes” were further asked the sources through which they heard of e-cigarettes, and the options included “Internet”, “TV”, “Newspaper”, “Outdoor advertisements”, “Family members, friends or colleagues”, and “Others”. Regarding the perception of e-cigarettes, the respondents were asked whether they believed that e-cigarettes were harmful to users and to others due to secondhand smoke, whether they believed that e-cigarettes would be addictive, and whether they agreed that e-cigarettes would be a safe replacement for conventional cigarettes. The answers included “Yes”, “No”, and “Don't Know”.

We measured the use of e-cigarettes with the question, “Do you use e-cigarette now?” The options were on a 1–4 scale and referred to lifetime frequencies, including “Often (more than 20 times)”, “Occasionally (<20 times)”, “Ever used 1–2 times”, and “Never used” (Trumbo & Kim, 2015). For those who ever used e-cigarette, no matter the frequency of their uses, we further asked them where they obtained the e-cigarettes, and the answers included “Bought from Internet vendors”, “Bought from licensed cigarette stores or other stores”, “Bought from TV shopping channels”, “Received as gifts”, and “Others”. We asked those who ever used e-cigarettes the reasons of using. Multiple choices were allowed, including, “I wanted to avoid the harm of cigarettes”, “I wanted to avoid the secondhand smoke harmful to others”, “E-cigarettes could help me quit smoking”, “E-cigarettes were cheaper than cigarettes”, “I could use e-cigarettes in smoke-free areas”, “Using e-cigarettes was fashionable”, and “Out of curiosity” (Jiang et al., 2016).

Finally, we analyzed the association between e-cigarette use and smoking abstinence trying behavior among the current smokers by conducting a logistic regression analysis. Smoking cessation trying behavior is a dummy variable, as explained earlier. We also created a dichotomous variable for e-cigarettes use by recoding those who reported having used e-cigarettes “Often (more than 20 times)”, “Occasionally (<20 times)”, and “Ever used 1–2 times” as “Yes” while keeping those “Never used” as the reference group. For the purpose of conducting regression analysis, we included a number of social demographic variables for control, including sex, education, economic status, employment status, and type of household registration (*hukou*). SAS 9.3 statistical software was used for all the above analyses.

## 3 Results

Among the 2042 adolescent respondents in our survey, 615 or 30.12% were current smokers, 192 or 9.4% were former smokers or those who had successfully quit smoking, and 1235 or 60.48% were never smokers. Among current smokers, 62.62% smoked daily while others smoked occasionally. Also, 57.10% of current smokers smoked 1–5 cigarettes/day, 27.44% smoked 6–10/day, 11.83% smoked 11–20/day, and 3.63% smoked >20 cigarettes/day. Table 1 shows the socio-demographic characteristics of the current, former and never smokers.

**Table 1:** Sample characteristics (*N* = 2042).

	All sample (N = 2042)	Current smokers (*N* = 615)	Former smokers (*N* = 192)	Never smokers (*N* = 1235)
	N (%)	N (%)	N (%)	N (%)
Sex				
Male	1706 (83.55)	552 (89.76)	163 (84.90)	991 (80.24)
Female	336 (16.45)	63 (10.24)	29 (15.10)	244 (19.76)

Economic status				
Very wealthy	198 (9.70)	119 (19.35)	23 (11.98)	56 (4.53)
Comfortable	611 (29.92)	173 (28.13)	54 (28.13)	384 (31.09)
Fair	1003 (49.12)	269 (43.74)	100 (52.08)	634 (51.34)
Difficult	143 (7.00)	40 (6.50)	10 (5.21)	93 (7.53)
Very difficult	87 (4.26)	14 (2.28)	5 (2.60)	68 (5.51)

Education level				
High school or under	1222 (59.84)	356 (57.89)	99 (51.56)	767 (62.11)
Junior college	608 (29.77)	206 (33.50)	69 (35.94)	333 (26.96)
College and above	212 (10.38)	53 (8.62)	24 (12.50)	135 (10.93)

Employment status				
Employed	756 (37.02)	284 (46.18)	81 (42.19)	391 (31.66)
Not employed	1286 (62.98)	331 (53.82)	111 (57.81)	844 (68.34)

Location and type of *hukou*				
Local/urban	656 (32.13)	217 (35.28)	55 (28.65)	384 (31.09)
Non-local/urban	178 (8.72)	68 (11.06)	24 (12.50)	86 (6.96)
Local/rural	995 (48.73)	268 (43.58)	101 (52.60)	626 (50.69)
Non-local/rural	213 (10.43)	62 (10.08)	12 (6.25)	139 (11.26)

Table 2 demonstrates the awareness, perception and use of e-cigarettes among Chinese adolescents. Nearly 90% of the respondents heard of e-cigarettes, while never smokers' awareness rate (85.75%) of e-cigarettes was significantly lower than that of current smokers (95.93%) and former smokers (93.23%). Chinese adolescents learned about e-cigarettes primarily through the Internet (44.47%), TV (28.17%), and family, friends or colleagues (18.11%). More current smokers (50.51%) than former smokers (44.69%) heard about e-cigarettes from the Internet, while more former smokers learned about e-cigarettes through TV, newspaper, and friends and others.

**Table 2:** Awareness, perception and use of e-cigarettes by smoking status.

	All sample (N = 2042)	Current smokers (N = 615)	Former smokers (N = 192)	Never smokers (N = 1235)	*X*^2^	*P* value
	N (%)	N (%)	N (%)	N (%)		
Awareness of e-cigarettes	1828 (89.52)	590 (95.93)	179 (93.23)	1059 (85.75)	48.5	<0.001

Sources of awareness (*n* = 1828)						
Internet	813 (44.47)	298 (50.51)	80 (44.69)	435 (41.08)	39.2	<0.001
TV	515 (28.17)	125 (21.19)	50 (27.93)	340 (32.11)		
Newspaper	31 (1.70)	13 (2.20)	7 (3.91)	11 (1.04)		
Outdoor advertisement	52 (2.84)	19 (3.22)	5 (2.79)	28 (2.64)		
Friends and others	331 (18.11)	98 (16.61)	32 (17.88)	201 (18.98)		

Perception of e-cigarettes (n = 1828)						
Not harmful to users	464 (25.38)	146 (24.75)	56 (31.28)	262 (24.74)	3.65	0.161
No secondhand harm	597 (32.66)	190 (10.39)	63 (3.45)	344 (18.82)	0.6	0.743
Not addictive	601 (32.88)	219 (37.12)	76 (42.46)	306 (28.90)	19.9	<0.001
Safe replacement for cigarettes	613 (33.53)	170 (28.81)	62 (34.64)	381 (35.98)	8.8	0.01

Use of e-cigarettes (n = 2042)						
Often (≥ 20 times)	130 (6.37)	94 (15.28)	14 (7.29)	22 (1.78)	603.1	<0.001
Occasionally (<20 times)	184 (9.01)	128 (20.81)	35 (18.23)	21 (1.70)		
Used 1–2 times	226 (11.07)	137 (22.28)	40 (20.83)	49 (3.97)		
Never used	1502 (73.56)	256 (41.63)	103 (53.65)	1143 (92.55)		

Sources of obtaining e-cigarettes (*n* = 540)						
Internet vendors	284 (52.59)	208 (57.94)	35 (39.33)	41 (44.57)	20.9	0.008
Licensed cigarette stores or other stores	150 (27.78)	88 (24.51)	34 (38.20)	28 (30.43)		
TV shopping channel	46 (8.52)	24 (6.69)	13 (14.61)	9 (9.78)		
Received as gifts	26 (4.81)	16 (4.46)	2 (2.25)	8 (8.70)		
Other sources	34 (6.30)	23 (6.41)	5 (5.62)	6 (6.52)		

Reasons of using e-cigarettes (n = 540)						
Avoid the harm of cigarettes	241 (44.63)	171 (47.63)	37 (41.57)	33 (35.87)	4.5	0.105
Avoid the secondhand harm	142 (26.30)	87 (24.23)	32 (35.96)	23 (25.00)	5.2	0.076
Help quit smoking	131 (24.26)	105 (29.25)	26 (29.21)	0 (0.00)	35.5	<0.001
Cheaper than cigarettes	47 (8.70)	28 (7.80)	11 (12.36)	8 (8.70)	1.9	0.393
Avoid smoking bans	42 (7.78)	27 (7.52)	11 (12.36)	4 (4.35)	4.1	0.126
Fashionable	83 (15.37)	49 (13.65)	14 (15.73)	20 (21.74)	3.7	0.158
Out of curiosity	138 (25.56)	83 (23.12)	22 (24.72)	33 (35.87)	6.3	0.043

A quarter of the respondents (25.38%) believed that e-cigarettes would not be harmful to users, and around one third of them believed that e-cigarettes would not generate second-hand harm (32.66%), would not be addictive (32.88%), and would be a safe replacement for cigarettes (33.53%). A higher percentage of current smokers (37.12%) and former smokers (42.46%) than never smokers (28.90%) believed that e-cigarette would not be addictive. More never smokers (18.82%) than current (10.39%) and former (3.45%) smokers perceived that e-cigarettes would generate secondhand harm to others.

Former smokers seem to hold more positive views of e-cigarettes than current smokers. For example, significantly more former smokers than current smokers believed that e-cigarettes would be no harmful to users, not addictive, and a safe replacement for cigarettes. But more current smokers than current smokers believed that e-cigarettes would generate no secondhand harm.

Over a quarter of the respondents (26.44%) had ever used e-cigarettes, although only 6.37% were frequent users (20 times and more), 9.01% used <20 times and 11.07% used once or twice. But among current smokers, 58.37% also had used e-cigarettes, suggesting that they were dual users at the time of the survey. Current smokers were also more likely to have used e-cigarettes than former smokers, while only 7.45% of never smokers had ever used e-cigarettes.

More than half of the e-cigarette users (52.59%) obtained e-cigarettes from internet vendors, while 27.78% bought from licensed cigarette stores or other stores. The same pattern is observed in other countries such as New Zealand and U.S., where, despite national regulations on selling ENDS devices as medical products, e-cigarettes are accessible from the Internet for many users (King, Patel, Nguyen, & Dube, 2015; Li, Newcombe, & Walton, 2015). Others in our survey also bought from TV shopping channels (8.52%), received e-cigarettes as gifts (4.81%), and obtained from other sources (6.30%). Similar to the patterns of learning about e-cigarettes, current smokers (57.94%) were significantly more likely to have obtained e-cigarettes from the Internet than former smokers (39.33%), while former smokers were more likely than current smokers to have obtained e-cigarettes from licensed stores and TV shopping channel. More never smokers (8.70%) received e-cigarettes as gifts from others than both current (4.46%) and former (2.25%) smokers.

Chinese adolescents picked up e-cigarettes because they wanted to avoid the harm of cigarettes (44.63%), or to avoid the secondhand harm of smoking to others (26.30%), or they expected e-cigarettes to help quit smoking (24.26%). Meanwhile, a significant portion of the respondents used e-cigarettes because they thought it was fashionable to do so (15.37%) or simply because they were curious of e-cigarettes (25.56%). A smaller number of e-cigarettes users appreciated that e-cigarettes were cheaper than conventional cigarettes or were handy wherever a smoking ban is applied. More current smokers (47.63%) than former smokers (41.57%) used e-cigarettes to avoid the harm of cigarettes, while more former smokers (35.96%) than current smokers (24.23%) used e-cigarettes to avoid the secondhand harm of cigarettes.

Both current and former smokers had used e-cigarettes as a method to quit smoking. As shown in Table 3, 483 out of 615 current smokers tried to quit smoking. More than one third (36.02%) of the quit triers used e-cigarettes to help quit, while the rest used patches (12.01%) and other methods (51.79%). Among the 192 former smokers or those who had successfully quit, 17.19% used patches, 13.54% used e-cigarettes, and 69.72% used other methods to help quit.

**Table 3:** Use of e-cigarette as a method of smoking cessation.

Methods used for quitting	Current smokers who have tried to quit (*N* = 483)	Former smokers (N = 192)
	N (%)	N (%)
E-cigarette	174 (36.02)	26 (13.54)
Patch	58 (12.01)	33 (17.19)
Other methods	251 (51.79)	133 (69.27)

Are those who had used e-cigarettes more likely to have tried to quit smoking? We have applied a multivariate logistic regression to further test the association between e-cigarette use and quitting behavior. As Table 4 shows, e-cigarette users did seem more likely to have tried to quit smoking than never users. The odds ratio of an e-cigarette user to have tried to quit smoking is 1.60, and the result is significant at the 0.05 level. None of the control variables have demonstrated significant association with quitting behavior. In order to asses the validity of the fitted regression model, we have tested the association of predicted probabilities and observed responses. The correlation indices show that the model does validly predict the response although the strength of association is moderate (c = 0.627). Finally, we are not only interested in finding whether e-cigarette use would have any effect on quitting attempts but also quitting itself. Unfortunately, in two separate regression tests (results not shown here), neither the use of e-cigarettes or the frequently of use has shown any statistically significant impact on quitting.

**Table 4:** Association of e-cigarette use and quitting behavior (*N* = 590).

Variables	OR (95% CI)
Use of e-cigarettes	
Yes	1.60 (1.01–2.53)⁎
Never	1.00
Sex	
Male	1.12 (0.54–2.32)
Female	1.00
Economic status	
Very wealthy	1.72 (0.43–6.87)
Comfortable	1.81 (0.49–6.70)
Fair	2.11 (0.58–7.62)
Difficult	1.13 (0.27–4.78)
Very difficult	1.00
Education level	
Junior college	1.01 (0.64–1.58)
College and above	0.69 (0.340–1.42)
High school or under	1.00
Employment status	
Employed	0.71 (0.46–1.08)
Not employed	1.00
Type of *hukou*	
Urban	0.93 (0.60–1.45)
Rural	1.00
Location of *hukou*	
Local	0.76 (0.47–1.24)
Non-local	1.00
Perception of e-cigarettes	
Not harmful to users	1.02 (0.56–1.86)
No secondhand harm	1.30 (0.74–2.28)
Not addictive	0.73 (0.46–1.17)
Safe replacement for cigarettes	0.71 (0.46–1.11)


Association of predicted probabilities and observed responses	
Concordant = 62.6% Discordant = 37.1% Tied = 0.3% (57,096 pairs)	Somers' D = 0.255 Gamma = 0.255 Tau-a = 0.084 c = 0.627

⁎ *p* < .05.

## 4 Discussion

This study is one of the first empirical research on the characteristics of e-cigarette awareness, perception and use, and on the relationship between e-cigarette use and smoking cessation behavior among Chinese adolescents. E-cigarettes are rather popular among Chinese adolescents. The awareness rate (89.52%) is comparable with the ones reported in other countries (86% in the U.S. and 75% in Hong Kong) (Jiang et al., 2016; King, Alam, Promoff, Arrazola, & Dube, 2013), while the usage rate (26.44%) is higher than the combined rate for both current and ever e-cigarette users reported in other countries (Czoli, Hammond, & White, 2014; S. Lee et al., 2014). The Internet provides easy access to the information and sales of e-cigarettes for current, former and never smokers, as seen from the high proportion of the respondents reporting their awareness and purchase of e-cigarettes from the Internet.

Like many studies of e-cigarette use that include adults (Adkison et al., 2013; King et al., 2013; J. A. Lee, Kim, & Cho, 2016), our study of adolescents shows that current smokers were more likely than former smokers to have used e-cigarettes. As both argued in other studies (Kralikova, Novak, West, Kmetova, & Hajek, 2013) and shown in our study, current smokers tend to use e-cigarettes to help quit smoking. Alternatively, the finding may indicate that e-cigarette use may not support quitting.

As shown in other studies, the real impacts of e-cigarette use on smoking cessation remains to be seen (Borderud et al., 2014). By looking at the motivations of e-cigarette use, nearly half of the smokers believed that e-cigarettes would help reduce the harm of smoking and a quarter of them believed that e-cigarettes would help them quit. Meanwhile, 36.02% of those who had tried to quit smoking used e-cigarettes to help, and 13.54% of the successful quitters used e-cigarettes to help. However, this does not mean that e-cigarette use did help them quit. What our study does demonstrate is that e-cigarette use may at least be associated with quitting attempts, as ever users of e-cigarettes were shown to be significantly more likely to have tried to quit smoking than never users.

In our study, never smokers (21.74%) were more likely than both current (13.65%) and former (15.73%) smokers to be appealed by the fashion of e-cigarettes, and they (35.87%) were also more likely than current (23.12%) and former (24.72%) smokers to use e-cigarettes out of curiosity. Existing studies do not seem to reveal much about the motivations of never smokers for using e-cigarettes, mostly because the sample size for never smokers who had used e-cigarettes usually was very small and researchers were not able to report the motivations of e-cigarette use for never smokers (Li et al., 2015). But our finding that adolescents who did not have a history of tobacco use but used e-cigarettes out of fashion or curiosity, raises the concern that they could be more susceptible to the marketing strategies of e-cigarette manufacturers who portrayed their products as fashionable and novel.

Our study also implicates that the trend of using e-cigarettes among adolescents may further grow as smokers tend to engage in e-cigarette promotion. For example, 46.10% of current smokers and 40.78% of former smokers had recommended e-cigarettes to others. Also, in the Chinese context, cigarette gifting and sharing has long been seen as an important way of maintaining social networking and relationships by smokers (Kohrman, 2007). The culture of cigarette gifting has promoted smoking behavior among Chinese youths (Wolfson, Forster, Claxton, & Murray, 1997). Our survey shows that the trend of e-cigarette gifting may have been developed, with 36.27% of current smokers and 29.05% of former smokers having bought e-cigarettes as gifts for others. A higher percentage of never smokers (8.7%) than current (4.46%) and former (2.25) smokers reported that the e-cigarettes they used were given as gifts by others.

This study is limited by a few drawbacks in the measurements of variables and in the instrument of data collection. First, the study would have benefitted from more precise measurements of certain variables. For example, the measurement of frequency of e-cigarette use did not specify the intensity. Having used e-cigarettes 20 times could mean 20 instances of a few puffs from an e-cigarette or 20 instances of using an entire e-cigarette. The ambiguity unintentionally left the question open to the respondents' interpretation and consequently affected the implications of our findings. Second, the mobile app-based survey did not allow us to strictly control the sampling process. A greater percentage of males were included in the sample. The number of former smokers captured in the sample was very small. Nonetheless, the large size of the sample and its coverage of various socio-demographic population groups in China offers us a rare chance of examining the status of e-cigarette use among Chinese adolescents and its association with smoking cessation behavior, which hopefully will put the issues in perspective for policymakers.

## 5 Conclusion

E-cigarettes are widely known and quite popular among Chinese adolescents. However, the association between e-cigarette use and smoking cessation deserves further examination until a clearer picture is revealed. As a regulatory system for the rapid development of e-cigarette industry is still missing in China, more empirical research is called for to help form evidence-based regulatory policy on e-cigarettes.

## Role of funding sources

This study was funded by grants from Bill & Melinda Gates Foundation (Grant # OPP1123586). The Gates Foundation had no role in the study design, collection, analysis or interpretation of the data, writing the manuscript, or the decision to submit the paper for publication.

## Contributors

XZ and XW designed the study and wrote the protocol. XX and YG collected the data. XZ and XW conducted the statistical analysis. XW and XZ wrote the first draft of the manuscript and all authors have contributed to and have approved the final manuscript.

## Conflict of interest

None declared.
